# Acceptance and challenges of the introduction of the routine community-based vitamin A supplementation strategy: the case of Burkina Faso

**DOI:** 10.1017/S1368980022000283

**Published:** 2022-05

**Authors:** Ousmane Ouédraogo, Augustin Nawidimbasba Zeba, Saidou Kaboré, Abdramane Berthé, Koiné Maxime Drabo, Dowrot Bertine Ouaro Dabiré, Estelle Bambara, Helene Ouédraogo, Tegawendé Pierre Ilboudo, Claudine Konaté, Denis Garnier, Gunter Boussery, Mediatrice Kiburente, Noël Marie Zagré

**Affiliations:** 1 UNICEF, Ouagadougou, Burkina Faso; 2 Health Sciences Research Institute, Burkina Faso; 3 Nutrition Directorate, Burkina Faso; 4 University of Dédougou, Dedougou, Burkina Faso; 5 Centre Muraz, Bobo Dioulasso, Burkina Faso; 6 UNICEF, Abidjan, Cote d’Ivoire; 7 UNICEF, Dakar, Sénégal; 8 UNICEF, Libreville, Gabon

**Keywords:** Acceptance, Adoption, Challenges, Routine community-based strategy, Vitamin A supplementation (VAS), Burkina Faso

## Abstract

**Objectives:**

With the phase-out of the polio campaigns, Burkina Faso has developed a new strategy for routine community-based vitamin A supplementation (VAS) by institutionalising community-based health workers (CBHW) to sustain the gain of two decades of successful programming. Formative research was conducted soon after the strategy was introduced to solicit feedback on the acceptability of the new approach by the implementing actors while identifying the main implementation challenges for improving its effectiveness and sustainability.

**Design:**

This qualitative study was conducted in 2018 through (i) document review, (ii) individual interviews with key informants at the central, regional and district levels, and (iii) focus groups with CBHW and caregivers.

**Setting:**

Data collection was carried out at six levels of sites covering the entire country and selected based on VAS coverage rates with the community routine. A total of six health districts were selected.

**Participants:**

We conducted 46 individual interviews with health workers and 20 focus groups with 59 CBHW and 108 caregivers.

**Results:**

The study showed good acceptability of the strategy by all stakeholders. In the first 2 years of implementation, the national coverage of VAS was maintained at a high level (above 90 %) and there was a reduction in operational costs. The main challenges included delayed CBHW remuneration and weak communication and supervision

**Conclusions:**

The acceptability of the community-based routine VAS was good and was perceived to have a high potential for sustainability. Addressing identified challenges will allow us to better manage the expectations of community stakeholders and maintain the initial results

Vitamin A (VA) deficiency is a risk factor for blindness and death from measles and diarrhoea in children aged 6 to 59 months^(1)^. VA is an essential nutrient for the maintenance of normal physiological function^(1)^. The lasting absence of a sufficient intake of VA or its precursors in a person results in increasingly serious disorders, ranging from functional disorders from low serum retinol (< 0·70 µmol/l) without any clinical eye symptoms such as night blindness^(2)^.

The fourth report on the world nutrition situation estimated that in 2000, between 2·8 and3·3 million preschool children were affected by clinical VA deficiency^(3)^.

In 2005, Africa and Southeast Asia were the geographic areas with the highest prevalence of serum retinol < 0·70 μmol/l among children under 5 years of age: 49·9 % in Southeast Asia and 44·4 % in Africa^(4)^.

More recently, analysis of data from 183 developing countries showed that between 1991 and 2013, the prevalence of VA deficiency, defined as a serum retinol concentration below 70 μmol/l, decreased from 39 % to 29 % and remains a major public health problem in developing countries, with sub-Saharan Africa and Southeast Asia still being the most affected regions^(5)^.

In Burkina Faso, partial data collected in the province of Sanmatenga in 2001 showed a prevalence of retinolemia of 84·5 % and 61·8 % in children under 5 years of age and their mothers, respectively^(6)^. Night blindness, the first clinical sign of deficiency, was found in 16 % of pregnant women in Gourma Province and even reached up to 52 % in some villages^(7)^. According to the 2003 Demographic and Health Surveys^(8)^, the prevalence of night blindness in women during their last pregnancies was 13 %, and the prevalence of ‘adjusted’ twilight blindness was 7 % in pregnant women, far above the norm of the WHO (< 5 %), and there is no indication that the situation has improved since. In fact, more recent data, although not covering the whole country, report that more than 40 % of schoolchildren in rural^(9)^ and urban^(10)^ areas had VA deficiency with serum retinolemia (< 0·7 μmol/l).

To cope with this situation, Burkina Faso, like several developing countries, has adopted biannual supplementation with VA capsules for children under 5 years of age as a strategy to fight VA deficiency. Starting in 1998, it was established in Burkina Faso under the name Vitamin A Days (VAD) and then extended and adopted in VAD-Plus by the Ministry of Health (MOH) as a national strategy for child survival in 2011. Activities were organised periodically to offer an integrated package of preventive services known for their excellent cost-effectiveness and aimed at improving the health and survival of the child.

The VAD-Plus aimed to cover all children under the age of 5 years once every 6 months over a specific week. The essential package of preventive health services is defined according to the circumstances and needs at the local level. Generally, the core content of the package includes VA supplementation (VAS), deworming, screening for acute malnutrition, and distribution and/or awareness of the use of impregnated mosquito nets; other services are selected and added based on needs per region.

VAD-Plus campaigns have long benefited from the financial and technical support of UNICEF, HKI and the Micronutrient Initiative (MI) (renamed Nutrition International; NI) since the VAD’s initiation in 1998 until 2008, and then from 2009 to 2014 received the support of the World Bank. Since 2015, when funding from the World Bank ended, UNICEF and HKI have secured funding while notifying and advising that VAD-Plus in the form of campaigns have become unsustainable due to funding constraints.

In this context of the scarcity of financial resources for the organisation of VAS campaigns, in 2017, the government of Burkina Faso seized the opportunity to recruit 16 000 community-based health workers (CBHW) in rural areas with the aim of strengthening community health services through a broader strategy by the state to cover and provide package services, including VAS. The integration of the VAS in this community package offers an alternative to supplementation campaigns. Two CBHW were recruited from each village and received training to deliver a package of activities, including routine VAS in rural areas, while no change was made in urban areas to the campaign mode of the distribution of VAS.

The new strategy for routine community-based VAS in rural areas was thus designed to be carried out for a period of 30 consecutive days, that is, 1 month, at the same time throughout the country and in conjunction with the provision of other community health services; this continued to be offered twice a year.

During the supplementation period, rural CBHW go door-to-door supplementing children in the 6- to 59-month age group with VA. In addition, children who are in the 12- to 59-month age group are dewormed. In urban areas, distribution agents are specifically recruited and paid for 4 d^(4)^. In addition, each health facility becomes a fixed post to cover those children who failed (or missed) to receive the VA supplement and/or the deworming tablet. Data are collected daily during the 4 d in the urban environment and weekly for the rural environment. The data are compiled at the health facility level before being communicated to the health district level, then to the Regional Health Directorate and finally to the national level. There is supervision of these interventions in both urban and rural areas.

The strategy of engaging rural CBHW as part of community health development started in 2017 (second round of supplementation) and was adopted with the hypothesis that this would allow costs to be reduced without a reduction of efficiency.

After two initial rounds of the community routine strategy in 2017 and 2018, the objective of this study was to analyse the implementation actors’ acceptability of the introduction of the new routine community-based VAS strategy in the context of rural Burkina Faso while identifying the main challenges for improving its effectiveness, efficiency and sustainability.

## Method

### Type and sites of the study

This was a qualitative study that used a review of documents, direct interviews with key informants and focus groups at the community level. The review of documents focused on the activity reports of UNICEF and the Nutrition Department of the MOH from 2015 to 2018, establishing the operational cost budgets of the VAD-Plus, the reports on the administrative coverage of the health districts, HKI-supported post-distribution coverage survey reports and VAS coverage data collected in national SMART nutrition surveys. We also consulted the minutes of meetings related to VAS as well as the reports of training provided to CBHW. Only administrative coverage reports and reports validated by the government were included. As far as the budgets are concerned, only the total expenses provided by the nutrition directorate under UNICEF and HKI funding for the operational costs covering the whole country were included. Not all expenses could be included in the study, notably the salaries of the CBHW and the local financial contributions of the management committees of the health facilities. The purpose of the direct interviews was to investigate in depth the acceptability and adoption of the strategy by the CBHW as seen by the key stakeholders in the health sector based on their experiences in the field (central managers, technical and financial partners, health workers, non-governmental organisations (NGO) managers) and the focus group participants (CBHW, mothers and fathers of children under 5 years of age).

Data collection was carried out at six levels that covered the entire country: (i) the central level (Ouagadougou), (ii) the regional level, with the Regional Health Directorates, (iii) the district level, with the health districts, (iv) the primary health centre level and (v) the village level. A sixth level of data collection was deemed appropriate to obtain additional information on the strategy in urban areas. The peripheral health facilities of the two districts of Bobo Dioulasso (Dô and Dafra) were included in the study.

### Selection procedures for the different study sites

Ouagadougou (the national capital) was included as a study site, since the initial concept and the design of the VAS strategy in a systematic community routine took place there. Therefore, information from central decision-makers and from financial and technical partners involved in implementing the strategy was available in Ouagadougou.

### Selection of Regional Health Directorates and health districts

The selection of health districts was carried out as indicated below:

- All the health districts of Burkina Faso were grouped into three strata according to the coverage rate of the first round of the VAS for the year 2017.
- Two health districts were selected from each stratum, that is, a total of six health districts for the study. Three strata were identified as follows:

Stratum 1: Health districts with a coverage rate of ≥100 %; the health districts of Ténado and Séguénéga were selected.

Stratum 2: Health districts with a coverage rate of < 99 % and ≥ 80 %; the health districts of Bogandé and Nouna were selected.

Stratum 3: Health districts with a coverage rate of <80 %; the health districts of N’Dorola and Koupela were selected for having the lowest coverage.

Regional Health Directorates corresponding to these six health districts were also selected.

### Selection of primary health facilities and villages

In each of the two districts identified at the stratum level, the two health areas selected were the health area with the highest coverage in the district and the health area with the lowest coverage in the district.

The health facilities corresponding to these health areas were selected for the survey. The capital villages of each of the selected health areas were then selected. The aim was to diversify the characteristics of the respondents according to their residence to guarantee a representation of opinions and to achieve saturation.

### Target populations and sampling procedures by study site

Table 1 provides a description of the target populations and the sampling method according to the different levels: central level, regional or district level, health facility level and village level.

**Table 1:** Target populations and sampling procedures

Level	Target populations and sampling procedures by study site
At the central level	This level included resource persons involved in the fight against VA deficiency at the level of the MOH (Directorate of Nutrition and Directorate in charge of community health), and technical and financial partners (UNICEF and HKI) involved in the implementation of the routine strategy or concerned by the issues of malnutrition in general or micronutrient deficiencies in particular.
At the level of a Regional Directorate or a health district	The Regional Director was systematically selected for the study, along with the District Chief Medical Officer of the selected district and with him the head of the nutrition service and two non-governmental organisations (NGO) intervening in the nutrition sector or intervening in the fight against VA deficiency, when applicable. Head nurses and distribution agents from health centres on the outskirts of Bobo Dioulasso were also selected.
At the health facility level	Health workers and CBHW were all involved in VA supplementation. Two health workers from the same health facility, namely, the head nurse and the person responsible for well-baby visits, were systematically selected. For the CBHW, at least eight of them were selected with the following conditions. The CBHW of the health facility headquarters village were systematically selected. At least four female CBHW (if present) were selected. Complementary CBHW were chosen in concert with the head nurse of the health facility with the aim of retaining those most involved in VA supplementation.
At the level of a village	The study targeted mothers and fathers of children under 5 years of age. In the villages, the two community-based health workers (CBHW) were asked to help the team put together a group of eight mothers and eight fathers of children under 5 years of age, making sure they all came from different neighbourhoods.

VA, vitamin A; MOH, Ministry of Health; CBHW, community-based health worker.

### Collection instruments according to site and target

A semi-structured interview guide was used for the individual interviews. We developed and used a focus group guide to conduct the following discussions:

- One focus group per health facility with the CBHW.
- One focus group per village with mothers of children under 5 years of age.
- One focus group per village with fathers of children under 5 years of age.
- One focus group with the head nurses of the health facilities on the outskirts of Bobo Dioulasso (urban comparison).
- One focus group with distribution agents of the health facilities on the outskirts of Bobo Dioulasso (urban comparison).

Each focus group consisted of eight people and the average duration was 45 min. Focus groups with CBHW and mothers were often done in local languages and led by trained interviewers. Interviews and focus groups were audio-recorded and transcribed by the interviewers.

#### Deployment and data collection

After a 3-d training including 2 d in the classroom and 1 d of pretesting, twelve investigators forming six mixed pairs were selected to conduct the data collection. The investigators were deployed by pair and by selected district. Each pair was deployed on its survey site no later than the day before the start of the study. Each pair had information on the people to be interviewed at their disposal.

In total, the data were collected in the geographic areas covered by six Regional Health Directorates, six health districts, six health facilities corresponding to the six chosen health districts, and six villages in each of the selected health facilities. The study was conducted from 27 August to 5 September 2018 in rural areas. It continued until 17 September in Ouagadougou and Bobo Dioulasso.

#### Supervision of data collection

The investigation team was composed of a nutritionist and a health socioanthropologist who supervised the data collection in the six districts to ensure that for each district, the targeted persons were informed and available for the investigation. On each site, the team ensured the proper conducting of the data collection by direct monitoring of the pairs deployed in the field and the quality control of the data.

#### Analysis plan

Data analysis was performed according to the study objectives to answer key questions for each aspect to be investigated. Data processing was performed on the basis of the analysis and interpretation of the content of the participants’ responses. To this end, the investigators proceeded to the synthetic transcription of the verbatim responses, the identification of key concepts and the identification of excerpts from responses illustrating important concepts and concepts, taking into account the objectives of the study. A first reading of interviews made it possible to develop an analysis plan composed of items. Each item was illustrated by excerpts from each category of informants.

## Results

### Profile of participants in direct interviews

In total, 46 individual interviews and 20 focus groups with 167 participants were conducted as part of this study (Table 2).

**Table 2:** Distribution of the profiles of the participants in the individual interviews and focus groups

Individual interviews		Focus groups	
Profile of the interviewees	*n*	Profile of participants	*n*
Regional health director	6	Focus group with CBHW	7
Chief medical officer	6	Focus group with mothers	7
Head nurses	10	Focus group with fathers	6
Head of the healthy infant consultation service	4		
Nutrition focal point	6		
NGO representatives	4		
Nutritionist from the Nutrition Directorate	02		
Director of Health Promotion Education	01		
Financial partners	03		
	46		20

CBHW, community-based health worker; NGO, non-governmental organisation.

### Knowledge of the new strategy

The frontline actors in the implementation of the new VA distribution strategy were the CBHW, and male and female CBHW alike affirmed to know it as: a distribution of two different doses of VA capsules, for two age groups of children: (i) children from 6 to 11 months old and (ii) children from 12 months to less than 5 years old, whose distribution was also associated with administration of an anti-parasite treatment. This distribution is combined with the screening for malnourished children. One of the CBHW put it this way, ‘*We are given to distribute to children, since it is intended for children, 6–11 months old and 12–59 months in households. There are two kinds of colours: red for children 12–59 months and blue for children 6–11 months*’.

The parents of children (fathers and mothers) as well as the community leaders interviewed were also familiar with the strategy. A father of a child under 5 years old expressed himself as follows in response: ‘*The CBHWs collect the VAs in the health facilities. When they arrive in our households, they explain to us first that these vitamins fight against malnutrition and promote the physical and mental development of the child. They tell us which children are affected. There are two types of VA that they present to us: for the little ones and for the slightly older ones. After this passage, they tell us to wait for them yet another time for supplementation. With the two times in the year… I would like to ask the state if it can increase to four times. Because it helps children in their development’.*

For all CBHW, not only is the strategy known, but it is positively and unanimously appreciated by all of the CBHW encountered in this study. The comments below clearly express this satisfaction of the actors interviewed. ‘*Yes. It is like I said it allows us to reach all children. Second, we can work at our own pace and better plan the work*’.

A mother of a child under 5 years of age added*: ‘The CBHWs collect the VAs from the health facilities. When they arrive in our households, they first explain to us that these vitamins fight malnutrition and promote the physical and mental development of the child. They tell us which children are concerned. There are two types of VA that they present to us: for the very young and for the slightly older ones. After this visit, they tell us to wait another while for the supplementation’*.

All the health workers interviewed from the central level to the operational level were familiar with the new routine VA distribution strategy. In addition, most health workers said that the VA distribution strategy made it possible to reach the maximum number of children in the shortest possible time. The implementation of the strategy was also, according to them, an opportunity for the CBHW to identify and refer to health centres all sick people encountered in households.

According to them, the involvement of the CBHW was relevant because of their proximity to the community and at the same time it alleviated the workload at the level of the health centres. One health worker put it this way: ‘*The work of the CBHWs removes another burden from health facilities that would have fallen on health workers. It helps, because CBHWs are a transmission belt from health facilities to the community. For example, if we have a patient who comes to us and says he comes from a given village, we already know the CBHWs serving in this village and we can call him or her to inquire about who we may need, so to catch up with this person it’s easier’.*

### Acceptability and adoption of the new strategy

Most CBHW said they accepted the new strategy because (i) the distribution of this VAS is part of their job description and (ii) the VAS has many benefits for children, parents and the community. Beyond these two reasons mentioned above, the fact that almost all the children targeted for supplementation were reached was, in their opinion, proof of their acceptance of the strategy. One of them said, ‘*We are happy with the work because we have seen satisfactory results on them (the children), it is beneficial. We are not working for ourselves; it is an effort for the community. We live with children, mothers as CBHW. If they are healthy, it is our joy. This is what strengthens us in our work to reach as many children as possible’.*

Parents of children under the age of 5 years as well as community leaders interviewed declared that the CBHW have accepted the strategy as demonstrated by their perceived commitment and the dedication with which they carried out their task. A community leader summed up this point in the following words: ‘*When it comes to courage, we thank God, for that they are really courageous. If there are not two kinds of courage, they are courageous. Why am I saying this? Walking through all the concessions and knowing all the children who live there is not easy. You don’t live in a concession (local community) and you have to know all the kids! Sometimes they cannot find some at home, they record their names and come back after to administer the medicine. In my opinion, courage does not go beyond that. When they meet a targeted child in the village, they find out if she or he has received the medicine by checking the finger for a mark, before administering the medicine. For me, they are brave because they go into all concessions* ’

Health workers from the operational level to the regional directorates felt that acceptance of the strategy by the CBHW was not a choice that was left to the CBHW, as long as they fulfilled the job description for which they had been recruited. For them, it was a matter of duty and accountability even though they recognised the need to keep their motivation and commitment high. Most of the CBHW were aware that changing the modality from a ‘supplementation campaign’ to ‘routine supplementation’ may uncover and initially lead to misunderstandings, but they tied them to the difficulties inherent to any change, which they believed would disappear over time.

In any case, the health workers affirmed that after the ‘door-to-door’ visits carried out by the CBHW, very few children seen in consultations at the health facility during this time were not supplemented with VA, which constitutes an indicator of the acceptance of this new strategy. A Regional Director of Health put it this way: ‘… *I think that overall, in the field, they agree to do it otherwise we would not have achieved these objectives (VA coverage rate). Me in my locality, the objectives at the level of all the districts have been achieved. At the last round of supplementation all the districts were able to achieve their objectives. So I still tell myself the job is well done at the base’*.

Community members, parents of children under 5 years of age and community leaders said they had accepted and adopted the new strategy and each for their own reasons.

Mothers were particularly satisfied with the ‘door-to-door’ distribution because it saves them time. The fathers and community leaders interviewed believed they had an important role to introduce these CBHW to their wives. A father of a child under 5 years puts it this way: ‘*No, there are no fathers who refuse; They are well informed that it greatly improves the health of children. We men explain to women that refusing to supplement children can in the long run lead to high expenses; You can end up spending thousands of francs that you don’t necessarily have; What to do if not go into debt which is not obvious; It is therefore important to supplement the children to avoid certain expenses; all men know this and this is what they do’*.

All community actors recognised the relevance of using fixed points (to gather those who missed the house-to-house supplementation) in that they allowed those absent during the distribution period to be able to benefit from VA.

### Challenges of accepting and adopting the new routine community-based strategy

For all of the people interviewed (CBHW, parents of children, community leaders and health workers), if the acceptance of the new strategy was almost unanimous, its adoption by CBHW still faced a certain number of challenges, the main one being ‘lack of financial incentive’ or ‘insufficient financial motivation’. Other challenges, such as the 1-month long duration of the strategy, a conflictual relationship between CBHW and the head nurse of the health facility, and occasionally poor reception of CBHW by beneficiaries at home, were also reported. The CBHW express themselves as follows on this question: ‘*1 month is better compared to 4 d in terms of coverage because in 4 d the children who are absent during our visit can no longer be caught; on the other hand if it is 1 month we have time to come back and catch up with them. But on the financial level the old system was more interesting because with the system of 4 d at the end of the campaign we were paid but with the 1 month modality, we are only given nothing under the pretext that it is part of our work. But we don’t have a salary either. It has been 8 months since we received a salary. So we are torn between the well-being of the population and our own survival’*.

‘*Some men do not accept that we enter their concession for VA supplementation, especially in urban areas, and this can be demotivating*’. The sustainability of the approach will also depend on the regular payment of the monthly salary by the state.

### Routine strategy just as effective and more cost-effective than supplementation campaigns

**Fig. 1:**
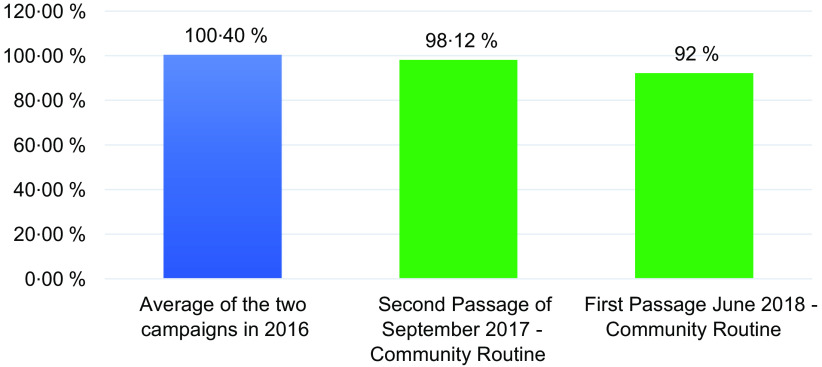
Comparison of national VAS coverage for children aged 6–59 months according to campaign mode *v.* community routine

We compared the coverage rate of the last VAD-Plus distribution campaign carried out in 2016 with those carried out with the new routine distribution strategy in 2017 and 2018. Figure 1 present coverage data. We found that coverage rates in the first 2 years (2017 and 2018) of community-based routine distributions were slightly lower than those of campaign mode distributions in 2016, although coverage rates remained above 90 %.

**Fig. 2:**
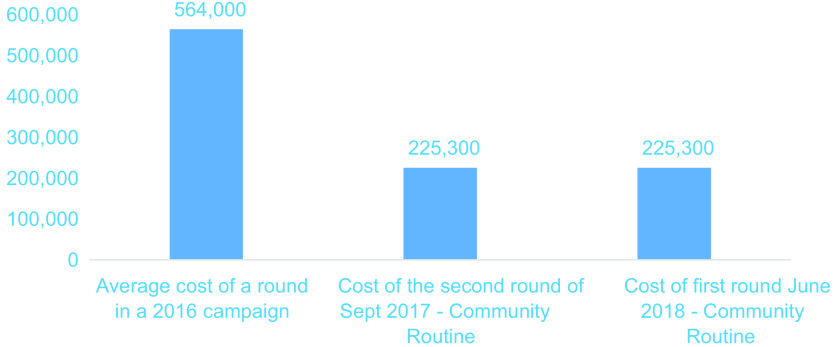
Budget comparison of operational costs (USD) according to campaign mode *v*. community routine

The operational cost of supplementation campaigns such as those of 2016 was approximately US $600 000 for a cost per child of approximately US $0·30, as presented in Fig. 2 below. The average operational cost of routine distribution of VA in 2017 and 2018 was US $225 000 for a cost per child of approximately US $0·12, that is, approximately 38 % of the 2016 VAS cost for that of the last two campaigns.

### The main challenges of introducing new routine VAS through community workers

Based on the individual and focus group interviews, the main challenges identified to strengthen the routine strategy were as follows:

- Communication around the routine strategy had not been optimal. It was noted that there was no national guide defining the different stages of the VAS new strategy.
- Supervision and close monitoring at the community level was not performed regularly
- Delays in payment were highlighted by CBHW. The sustainability of the monthly payment of CBHW by the state was not secured.

## Discussion

This article highlights a major programmatic innovation, which is the new community-based routine strategy for VAS as implemented in Burkina Faso. It provides an opportunity to compare this strategy with the campaign mode in Burkina Faso, as well as other routine VAS strategies implemented in other countries in Africa.

The distinguishing feature of the new routine strategy in Burkina Faso is the ‘door-to-door’ approach linked to the fact that CBHW reach children within households, which is comparable to the routine strategy implemented in Ethiopia^(11)^. However, this strategy is different from that reported by Horton *et al.* in Senegal^(12)^, where beneficiary children were supplemented in health structures, requiring them to travel to these health centres.

The evaluation of the acceptability and uptake of this strategy revealed a positive appreciation of ‘door-to-door’ distribution not only by parents of children under 5 years of age, especially mothers, on the grounds that it enabled them to save time for their activities. It also prevents disputes between women queuing during VAS when and if they were to all meet at a fixed place. Additionally, women do not need to face the difficulties of travel and stigmatisation (of those with several children), as has been reported in Ethiopia^(11)^ for mothers with more than one child in the 6 to 59 months age group.

This is an important factor of the acceptance of the strategy by the beneficiaries and may explain the average coverage rate above 90 %.

National coverage has been maintained at a good level above 90 % with the transition to community routine modality, according to reports from facilities. However, post-distribution coverage was 76·1 % (72·7–79·2 %) according to the results of the 2017 SMART National Nutrition Survey, compared to 97·9 % (97·4–98·4 %) found at the post-campaign coverage conducted in 2016. These differences in coverage or mismatches between the administrative data from the health facilities and the survey data could be related to: i) the quality or reliability of the administrative data reported during the VAD-Plus being questioned; (ii) the low level of control of the target populations in terms of projections in the absence of a real recent census; and (iii) possible memory bias among the women interviewed in the post-coverage studies. This situation recommends the strengthening of data validation reviews of the VAD-Plus for quality assurance. This coverage rate is slightly lower than those observed during supplementation campaigns in previous years in Burkina Faso, similar to those in Ethiopia, as also reported by Gatobu *et al.* in 2017^(11)^. Most mothers of children under 5 years of age, fathers and community leaders were in favour of the involvement of door-to-door visits by CBHW in VAS and saw in this an interest for the health of their children and their community in general. The involvement of the CBHW, who is a community member, reinforces this feeling of ownership of the strategy and constitutes a guarantee of good performance because the CBHW are from the community and know the eligible children better. We can also think that this knowledge of children within the community would increase over the years and should strengthen the coverage of routine distributions.

The coverage rate of the new routine community-based strategy by the CBHW observed in this study, although it approaches the rate of routine coverage implemented in Ethiopia^(11)^, is higher than the coverage rate of routine supplementation in Senegal^(12)^ and those of the Uganda supplementation campaigns^(13,14)^.

The impact of the change from 4 d to 1 month has resulted in a decrease in the workload of the CBHW; they are no longer under pressure compared to the 4-d mode, and it allows them to catch up with the children who are absent. However, a specific study on the workload of CBHW is needed due to the increasing number of programmes that rely on them. For health workers, the use of CBHW is of particular importance because it relieves the burden on health facilities; it also allows CBHW to refer to health centres people met in households during their visits who would have gone to health facilities too late or not at all. Unlike supplementation campaigns, ‘door-to-door’ distribution in households is personal and makes it possible to be more confident about the coverage rates reported; according to these health workers, after the distribution period, only a few children are non-supplemented when attending the health facilities. In addition, once this database of affected children is established, it can serve as a basis for the following years; children who have newly reached minimum age can be added, and those who leave the age group can be removed.

VAS is only a part of the job of CBHW; however, it is accepted and experienced as a duty they perform for the benefit of their community. Their role in supplementation does not involve a per diem payment, as was the case in the past with supplementation campaigns, and this probably explains the 62 % reduction in the operational cost during routine supplementation compared to supplementation in the campaigns in previous years. In addition, the operational cost of routine community supplementation estimated at approximately US $0·12 per child in Burkina Faso is lower than the cost per child estimated in other routine distributions, such as in Senegal and Ethiopia^(11,12)^, or in a campaign, such as the one conducted in Uganda^(13,14)^. It should be emphasised, however, that this cost is not only for VA but also for the other services offered at the same time (identification and referral of cases of illness, deworming, counselling, screening for malnutrition, etc.) as well as the positive effects in terms of community organisation, satisfaction, and so on.

This cost is lower than the costs of previous supplementation campaigns conducted in Burkina Faso and those reported elsewhere in Africa^(14–16)^.

However, one of the main weaknesses of this budget analysis is that it does not include all of the cost elements, particularly the salaries of the CBHW but also other local expenses borne by the management committees of the health facilities that are not documented at the central level. In view of this limitation, we recommend a specific study devoted to the analysis of the costs of the different VAS modalities.

Most of the CBHW recruited from the villages are those who in previous years were involved in supplementation in the form of campaigns and received per diem payment for that. This arguably explains why most of these CBHW considered the routine supplementation ‘unmotivated financially’ and cited this as a weakness of this strategy. Indeed, although recruited with a monthly financial motivation for a job that includes VAS, the previous habits of per diem compensation may give the CBHW the feeling of not being paid for an activity that they lead. This deserves to be addressed quickly because it constitutes a weakness that in the long term, according to the CBHW themselves, could hamper the adoption of the strategy. In addition, one of the challenges of sustainability is related to the regulation of the payment of the monthly salaries of the CBHW by the state. Despite this challenge, coverage rates remained above 90 %, higher than those observed in other supplementation experiments in routines or in the form of campaigns, and there was a much lower operational cost^(11,12,14,16)^.

The use of CBHW still faces several challenges, which will have to be overcome to sustain the original routine strategy and which seem to be more cost-effective than those reported in other countries. In fact, similar to the situation in Ethiopia, CBHW in Burkina Faso often must travel long distances to reach agricultural villages to supplement the children. They also face an unequal workload from village to village, as the recruitment of two CBHW per village does not consider the size of these villages. The results of the acceptability and adoption assessment revealed the reluctance of some heads of household to see the intrusion of men into the community as favourable. To prevent these incidents, the conditions for recruiting CBHW provide for perfect gender parity, which unfortunately cannot be achieved in several villages because of the minimum level of education required, often to the disadvantage of women. In fact, in most villages, there were few female CBHW.

While in rural areas the solution seems to have been found with the routine community-based strategy through the CBHW, this is not the case for urban areas where the state has not recruited CBHW. It is therefore necessary to continue to identify an alternative method to improve coverage in urban areas. Indeed, the results of the national coverage survey conducted in 2018 showed a higher VAS coverage rate in rural areas (77·3 %) than in urban areas (44·3 %).

Hence, there is an urgency to think about an innovative strategy of cocreation with beneficiaries to strengthen coverage in urban areas through implementation research^(17)^.

### Policy and programme implications for programme planners and implementers

As a child survival intervention, VAS is part of a package of services that comprises other child survival interventions such as deworming, screening and nutrition counselling and is provided by CBHW.

In view of the challenges observed, we propose the following strategies to improve the quality of the implementation of a routine community-based VAS strategy in Burkina Faso:

- Strengthening the community health strategy on which the routine strategy is based (capacity building and community information system).
- Strengthening the awareness/communication aspects of proximity and mass on VA.
- Strengthening social mobilisation around VAS (with the involvement of community and municipal leaders).
- Increasing the number of CBHW to more than two for large villages with populations above a certain threshold and addressing the insufficiency of CBHW, particularly in certain farming hamlets.
- Development of a VAS guide explaining the new strategy
- Continuing reflection to find a sustainable alternative for urban areas without community agents

## Conclusion

The results presented highlight the relevance of the VAS’s routine community distribution strategy in Burkina Faso, which benefits from the opportunity to recruit CBHW dedicated to strengthening community health. After 2 years of experience, the results are in favour of an acceptance of the strategy and a tacit adoption process that should be reinforced by more active communication with the various actors in the field.

The comparison of the operational costs of the 2 years of experience of implementing the routine community strategy with the last 2 years of supplementation in the form of a campaign shows a reduction of almost 62 % in cost per child in favour of the routine and a maintenance of the coverage rate greater than 90 %.

However, a specific study on budget analysis is needed to further evaluate cost-effectiveness to ensure that all generated operational expenses are taken into account. It is also necessary to recognise other non-calculable benefits of the routine, such as the improvement of the health of populations by screening for different diseases and the relief of health service burdens, which the use of CBHW allows.

This strategy remains an innovation for the fight against VA deficiencies. Taking measures to face the challenges identified would make it possible to strengthen the effectiveness of this routine community strategy and make it more sustainable.

However, additional implementation research would be important for further reflection to also identify a sustainable alternative for urban areas lacking community agents.
